# The Impact of Isotretinoin on Lipid Profile: a Systematic Review

**DOI:** 10.1097/MS9.0000000000003366

**Published:** 2025-05-20

**Authors:** Thiyagarajan Sibi Krishna, Ravneet Kaur, Vani Malhotra, Zaineb Fatima, Manahil Mustajab, Tarundeep Singh, Mahrukh Iqbal, Trisha Kesavan, S.P Ajeya, S. Grace Mathew, Mansi Singh

**Affiliations:** a Department of Medicine, University of Perpetual Help System Dalta, Rizal, Philipines; b Department of Medicine, Lady Hardinge Medical College, New Delhi, India; c Department of Medicine, Jawaharlal Nehru Medical College, Wardha, India; d Department of Medicine, Dow University of Health Sciences, Karachi, Pakistan; e Department of Medicine, Government Medical College, Patiala, India; f Department of Medicine, Karachi Medical and Dental College, Karachi, Pakistan; g Department of Medicine, SRM Medical College Hospital and Research Centre, Tamil Nadu, India; h Department of Medicine, Indira Gandhi Medical College and Research Institute, Puducherry, India; i Indira Gandhi Medical College and Research Institute, Bulgaria; j Department of Medicine, O O Bogomolets National Medical University: Nacional’nij medicnij universitet imeni O O Bohomol’ca, Kyiv, Ukraine

**Keywords:** lipid metabolism, cholesterol levels, dyslipidemia, isotretinoin, lipid parameters, lipid profile, lipoprotein levels, triglycerides

## Abstract

**Background:**

Isotretinoin, or 13-cis retinoic acid, is prescribed to treat moderate to severe recalcitrant nodulocystic acne that remains untreated with other drugs, such as antibiotics. The adverse effect profile of isotretinoin has raised concerns since studies have reported a substantial elevation in the low-density lipoprotein (LDL)/high-density lipoprotein (HDL) ratio, indicating the risk of cardiovascular diseases after a long-term course of isotretinoin. Assessing the impact of isotretinoin on lipid profile markers in acne patients was the aim of this systematic review.

**Method:**

Various databases, such as PubMed, Google Scholar, SCOPUS, Embase, and Web of Science, were comprehensively searched to identify relevant clinical studies. Ten articles out of 256 were selected based on the inclusion and exclusion criteria by two independent reviewers. Each review was thoroughly evaluated using Assessment of the Methodological Quality of Systematic Reviews and the Newcastle-Ottawa Scale.

**Key results:**

A decrease in HDL was observed, whereas total cholesterol, triglyceride, and LDL levels were notably elevated. However, most changes in lipid profile parameters are non-progressive, and their clinical significance is poorly understood. Liver enzyme levels, including aspartate transaminase and alanine transaminase, were altered to a lesser degree.

**Conclusion:**

Long-term use of isotretinoin is associated with mild alterations in the lipid profile, resulting in increased total cholesterol, triglyceride, and LDL levels. However, since lipid alterations vary depending on factors such as the population studied, dosage, and duration of isotretinoin treatment, regular monitoring of the lipid profile along with low density lipoprotein is recommended to avoid potential risk factors of isotretinoin on lipid metabolism and, thereafter, on the cardiovascular system, but is not deemed paramount.

## Introduction

Isotretinoin, known chemically as 13-cis retinoic acid, Accutane, or Roaccutane, is administered orally as a capsule in the treatment of refractory nodulocystic acne, ranging from moderate to severe ^[1,2]^.

**HIGHLIGHTS**

- Systematic review evaluating the impact of isotretinoin therapy on lipid profile parameters.
- Comprehensive synthesis of evidence from randomized controlled trials, observational studies, and meta-analyses.
- Analysis elucidates the relationship between isotretinoin treatment and lipid profile alterations.
- Findings inform clinical decision-making and patient counseling regarding isotretinoin therapy for severe acne management.

The exact mechanism of action of isotretinoin is unknown; however, it inhibits hyperkeratinization and comedone formation in patients with acne. It also induces apoptosis in sebaceous glands, which in turn leads to decreased sebum production^[3]^. This altered microenvironment within the duct suppresses Propionibacterium acnes, causing a drastic reduction in acute inflammation^[4]^. In addition, it also has an immunomodulatory action^[5]^.

Isotretinoin is prescribed predominantly for treating various forms of acne, specifically in severe cases that show no improvement after a course of antibiotics^[6]^. Isotretinoin has also proven to be useful in many other dermatological conditions, including psoriasis, rosacea, keratosis, and others^[7]^. Isotretinoin is less commonly used in the treatment of malignancies, such as T-cell lymphomas^[8]^ and neuroblastomas^[9]^.

The adverse effects of isotretinoin are a significant barrier to its use. The adverse effects include dry lips which was the most common to be reported by every participant, followed by xerosis, facial erythema. Almost a quarter of the patients also reported to have psychiatric symptoms^[10]^. Studies also show a significant decrease in the serum level of folic acid after 30 days of isotretinoin supplementation^[11]^. Lower back pain is also a common complication of isotretinoin administration^[12]^.

The levels of different lipids (fats) in the blood, such as total cholesterol, triglycerides, low-density lipoprotein (LDL) cholesterol, and high-density lipoprotein (HDL) cholesterol, are measured by a blood test called a lipid profile. These lipid levels can help assess an individual’s risk of developing cardiovascular diseases such as heart disease and stroke^[13]^.

The normal ranges of the lipid profile parameters were as follows.

1. Total cholesterol: < 200 mg/dL (desirable)^[14]^

2. LDL cholesterol: < 100 mg/dL (optimal)^[15]^

3. HDL cholesterol: > 40 mg/dL for men and >50 mg/dL for women (protective against heart disease)^[14]^

4. Triglycerides: < 150 mg/dL (normal)^[15]^

It is noteworthy that there may be modest variations in these normal ranges based on demographics like age, gender, and ethnicity^[16]^.

While initiating synthetic retinoid treatment, inadequate attention has been paid to the atherogenic effect and the risk it poses for coronary heart disease. With prolonged use of isotretinoin, studies have shown a substantial elevation of the LDL/HDL ratio of 0.92 ± 0.51, indicating the risk of cardiovascular disease. Patients with familial hypercholesterolemia are more susceptible^[17]^. Isotretinoin increases Apo C-III expression in human hepatoma HepG2 cells, the target gene contributing to the hypertriglyceridemia and atherogenic lipid profile following retinoid therapy^[18]^.

Given the rising concerns regarding the long-term effects of isotretinoin on lipid parameters, it is imperative to assess and monitor the lipid profiles during isotretinoin therapy. Lipids play a crucial role in various physiological processes. Altered lipid metabolism can adversely affect cardiovascular health, liver function, and overall lipid homeostasis. This study aims to investigate the effects of isotretinoin on lipid profiles and metabolism, providing valuable insights into the need for lipid assessment and monitoring during isotretinoin treatment to ensure patient safety and optimize therapeutic outcomes.

## Methodology

The review was registered on PROSPERO (registration number CRD42023428164)^[19]^ and was reported in line with the PRISMA criteria^[20]^ and is fully compliant with AMSTAR 2 criteria^[21]^.

### Search strategy

A comprehensive literature search for articles published between 2013 and 24 May 2023 was conducted using electronic databases, including PubMed, Embase, Google Scholar, SCOPUS, and Web of Science.

The initial search was conducted on 20 May 2023, and was updated and revised on 25 May 2023. It included the following terms: “Isotretinoin,” “lipid profile,” “cholesterol levels,” “triglycerides,” “dyslipidemia,” “lipid metabolism,” “lipid parameters,” “lipoprotein levels,” “HDL cholesterol,” “LDL cholesterol,” “Accutane,” and “Roaccutane.” No search limits were applied in this study. Additional sources, such as reference lists of relevant articles and clinical trial registries, were searched^[1]^. No language restrictions were applied.

### Study selection

**Figure 1.**
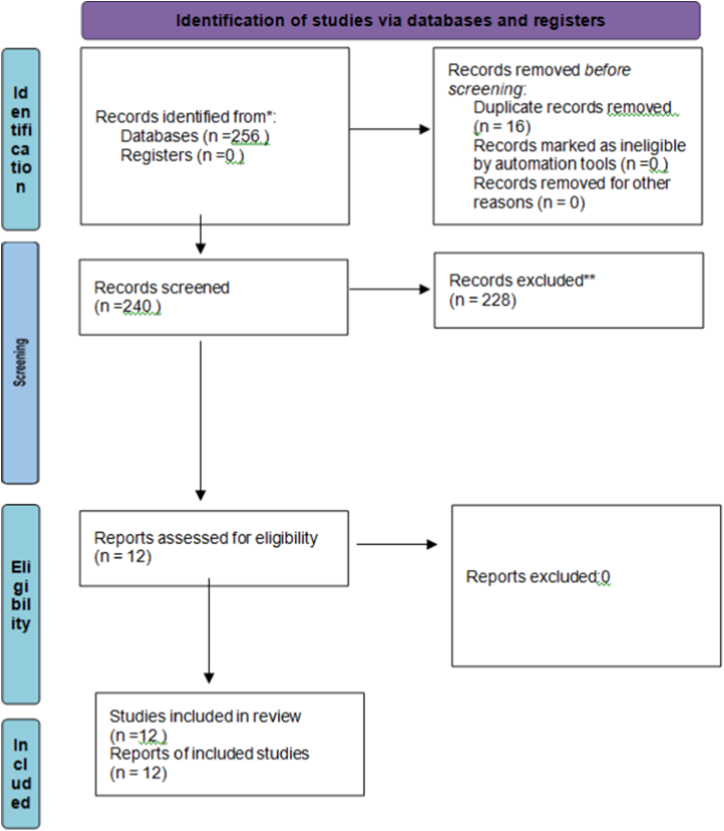
The PRISMA 2020 statement: An updated guideline for reporting systematic review.

Two independent reviewers, RK and TSK, screened 256 articles for the titles and abstracts of the identified studies for relevance. Screening was performed by reading the titles and abstracts and assessing full-text articles deemed relevant. A total of twelve articles were selected for this study. All relevant papers were read in full text by RK and TSK, and any discrepancies in study selection were resolved through discussion between all authors by consensus in accordance with the PRISMA (Preferred Reporting Items for Systematic Reviews and Meta-Analyses) Guidelines (The PRISMA 2020 statement: An updated guideline for reporting systematic reviews)^[20]^ as shown in Fig. 1.

Studies that involved human participants were included. Studies evaluating the impact of isotretinoin treatment on lipid profiles and participants with acne or other dermatological conditions receiving isotretinoin treatment were also included. Studies reporting quantitative data on lipid parameters such as total cholesterol, LDL cholesterol, HDL cholesterol, and triglycerides were also included.

Animal and in vitro studies were excluded. Studies that did not focus on the effect of isotretinoin on lipid profiles and those with a sample size less than a predetermined threshold were also excluded. Studies with insufficient data on lipid profile outcomes, case reports, reviews, commentaries, or editorials were also excluded.

### Data extraction

Three reviewers independently extracted data using a standardized form. Relevant information, including the author’s name, year of publication, and study design, was extracted from each study. Participants’ demographics were also extracted. Details of isotretinoin treatment were recorded, including the dosage and duration of treatment. The primary outcomes of interest were lipid profile parameters, including triglyceride, HDL, LDL, and total cholesterol levels. Whenever possible, data were extracted at different time points during isotretinoin treatment. Adverse events related to changes in lipid profiles were also documented. Any disagreements during data extraction were resolved with the involvement of other authors to ensure accuracy.

### Quality assessment

All twelve reviews selected based on the inclusion and exclusion criteria were rigorously evaluated using a modified version of the AMSTAR (Table1) and NOS^[22]^.

**Table 1:** AMSTAR checklist of all the included studies

Author	Yaldiz *et al*	Leelambika *et al*	Park et al	Alajaji et al	Al-haddab *et al*	Khabour et al	Lee et al	Hansen et al	Sarkar et al	Saklamas *et al*
1. Did the research questions and inclusion criteria for the review include the components of PICO?	N	N	N	N	N	N	Y	N	Y	N
2. Did the report of the review contain an explicit statement that the review methods were established prior to the conduct of the review and did the report justify any significant deviations from the protocol?	N	Y	N	Y	Y	N	Y	N	N	N
3. Did the review authors explain their selection of the study designs for inclusion in the review?	N	N	N	Y	Y	N	Y	N	Y	N
4. Did the review authors use a comprehensive literature search strategy?	N	N	N	N	Y	N	Y	N	N	N
5. Did the review authors perform study selection in duplicate?	Y	Y	N	N	N	N	Y	N	N	N
6. Did the review authors perform data extraction in duplicate?	N	Y	N	Y	Y	N	Y	N	Y	N
7. Did the review authors provide a list of excluded studies and justify the exclusions?	N	N	Y	Y	Y	N	Y	N	Y	N
8. Did the review authors describe the included studies in adequate detail?	Y	Y	N	N	Y	N	Y	N	Y	N
9. Did the review authors use a satisfactory technique for assessing the risk of bias (RoB) in individual studies that were included in the review? RCT	Y	N	N	Y	N	N	Y	N	Y	N
10. Did the review authors report on the sources of funding for the studies included in the review?	N	N	N	Y	N	N	Y	N	Y	N
11. If meta-analysis was performed did the review authors use appropriate methods for statistical combination of results? RCT	N	Y	-	N	Y	N	N	N	N	N
12. If meta-analysis was performed, did the review authors assess the potential impact of RoB in individual studies on the results of the meta-analysis or other evidence synthesis?	N	N	-	N	N	N	N	N	N	N
13. Did the review authors account for RoB in individual studies when interpreting/discussing the results of the review?	Y	N	-	N	Y	N	Y	N	Y	N
14. Did the review authors provide a satisfactory explanation for, and discussion of, any heterogeneity observed in the results of the review?	Y	Y	Y	Y	Y	N	Y	N	Y	N
15. If they performed quantitative synthesis did the review authors carry out an adequate investigation of publication bias (small study bias) and discuss its likely impact on the results of the review?	Y	Y	N	Y	Y	N	Y	N	Y	N
16. Did the review authors report any potential sources of conflict of interest, including any funding they received for conducting the review?	N	N	N	N	N	N	Y	N	N	N

## Results

The review was registered on PROSPERO (registration number CRD42023428164). All the studies and their characteristics have been summarized in Table 2. In a study by Saklamaz *et al*, isotretinoin treatment significantly increased lipid levels, Carotid Intima-Media Thickness (CIMT) (0.60-0.74 mm; P ˂0.001), whereas it non-significantly increased HOMA-IR (0.91-1.87; P = 0.70), OPN (4.32-5.44 ng/ml; P = 0.27), and hs-CRP (0.08-0.09 mg/dL; P = 0.88) levels. There was no correlation between OPN and CIMT (P = 0.77), suggesting that isotretinoin treatment for 4 months significantly increased CIMT in acne patients.

**Table 2:** Summary of studies investigating the impact of isotretinoin on lipid profile

Title	Year of publication	Study design	Participants	Intervention	Outcomes measured	Results	Conclusions
Isotretinoin increased Carotid Intima-Media Thickness (CIMT) in acne patients	2016	Prospective cohort study	21	Isotretinoin at a dose of 0.5-0.8 mg/kg for 4 months.	CIMT Osteopontin (OPN) Levels Lipid Levels High Sensitive C Reactive Protein (hs-CRP) Levels Insulin Resistance (HOMA IR)	CIMT (0.60-0.74 mm; P < 0.001); HOMA-IR (0.91-1.87; P = 0.70), OPN (4.32-5.44 ng/ml; P = 0.27), and hs-CRP (0.08-0.09 mg/dL; P = 0.88). There was no correlation between OPN and CIMT (P = 0.77).	Isotretinoin treatment for 4 months significantly increased CIMT in acne patients.
Evaluation of Lipid profile levels in acne vulgaris on low-dose isotretinoin: A prospective study	2016	Prospective cohort study	50	20 mg Isotretinoin daily for 4 months	Total Cholesterol Triglycerides HDL LDL	Statistically significant increase in cholesterol, triglycerides, and LDL, a significant decrease in HDL levels.	Intermittent isotretinoin therapy affects lipid profile, but the adverse effects can be treated without interrupting the treatment.
Optimal laboratory testing protocol for patients with acne taking oral isotretinoin	2023	Retrospective chart review	30	Varied with individual (total prescribed dose was a range of 600-3600 mg, total prescription days was in the range of 60-180 days)	AST, ALT, TG, HDL, LDL Total cholesterol	AST, ALT, TG, and HDL levels significantly changed between 5th and 6th months. However, LDL and total cholesterol levels significantly changed between 1st and 2nd months.	The study recommends testing AST, ALT, and TG levels once every 5 to 6 months and LDL and total cholesterol during the 1st and 2nd month on Isotretinoin as they are subject to change.
Laboratory Abnormalities in Acne Patients Treated With Oral Isotretinoin: A Retrospective Epidemiological Study	2021	Retrospective chart review	407	Unspecified dosage of Isotretinoin for a minimum of 4 months	TG, AST, ALT total cholesterol	AST and ALT levels were increased in 5.4% and 12.7% of patients, respectively, 12.7% of patients had increased triglyceride levels and 9% had increased cholesterol levels during their most recent visit.	Oral isotretinoin can cause an elevation in ALT, AST, total cholesterol, and triglyceride levels, but the incidence of these laboratory abnormalities is low, and the elevation was not associated with significant morbidity.
Association between leptin gene rs7799039 polymorphismand lipid profile changes induced by isotretinoin treatment in acne patients	2018	Cross-sectional study	200	Oral Isotretinoin therapy (40 mg/day) for at least 30 days	HDL, LDL, Total Cholesterol, AST, ALT at Baseline and after Treatment	Significant increase in the lipid profile (LDL, TC, TG), liver enzymes (AST, ALT) (*P* < 0.05). Levels of HDL significantly decreased after therapy (*P* < 0.05). Changes were linked with the genotypes and polymorphism of gene rs7799039.	Leptin gene polymorphism might modulate lipid parameters and liver enzymes of oral isotretinoin therapy among acne patients.
Laboratory Monitoring During Isotretinoin Therapy for Acne A Systematic Review and Meta-analysis	2016	Systematic review and meta analysis		Oral isotretinoin therapy (40 mg/day or more) for a period of 4 weeks	Laboratory values for Lipid profile, hepatic function and complete blood cell counts	Mean (99% CI) values during treatment (non-baseline) for triglycerides—119.98 mg /dL (98.58-141.39 mg/dL); for total cholesterol, 184.74 mg/dL (178.17-191.31 mg/dL); for low-density lipoprotein cholesterol, 109.23 mg/dL (103.68-114.79 mg/dL); for high-density lipoprotein cholesterol, 42.80 mg/dL (39.84-45.76 mg/dL); for aspartate aminotransferase, 22.67 U/L (19.94-25.41 U/L); for alanine amino-transferase, 21.77 U/L (18.96-24.59 U/L); for alkaline phosphatase, 88.35 U/L (58.94-117.76 U/L); and for white blood cell count, 6890/L (5700/L-8030/ ML).	Although isotretinoin is associated with statistically significant changes in the mean values of several laboratory tests, the mean changes did not meet a priori criteria for high risk. Therefore, evidence from this study does not support monthly laboratory testing for standard doses of oral isotretinoin for standard patients with acne.
Standardized laboratory monitoring with use of isotretinoin in acne	2016	Retrospective view of laboratory data	515	Low-dose isotretinoin	• Leukopenia • Thrombocytopenia • Elevated Liver Transaminases • Triglyceride Levels • Cholesterol Levels	Leukopenia—1.4% and Thrombocytopenia in 0.9% patients. Elevated Liver enzymes—1.9% vs 1.6% at baseline. Triglycerides and cholesterol—significant elevations in 19.3% and 22.8%, respectively. Mean duration of treatment before abnormalities detected- hypertriglyceridemia-56.3 days, ALT-61.9 days, Hypercholesterolemia-50.1 days	In healthy patients with normal baseline lipid profiles and liver function tests, repeated monitoring of the above parameters should be performed after 2 months of isotretinoin therapy, and if the results are normal, no further testing is required.
Assessment of auditory function and lipid levels in patients receiving oral isotretinoin (13-cis retinoid) therapy for acne vulgaris.	2018	Prospective cohort study	40	Isotretinoin therapy (0.5 g/kg)	• Distortion Product Otoacoustic Emissions (DPOAEs) • Pure Tone Audiometry Threshold • Aspartate Aminotransferase (AST) • Alanine Aminotransferase (ALT) • Total Cholesterol • Triglyceride • High Density Lipoproteins (HDL) Cholesterol Levels • Low Density Lipoproteins (LDL) Cholesterol levels	Mean PTA value after treatment = 6.5 ± 3.5. Mean value for the DPOAE test- No statistically significant difference was observed at any frequency. ALT levels after treatment 13.4 ± 4.7 (*P* value = 0.02). AST levels after treatment = 18.2 ± 3.6 (P value = 0.01). Total cholesterol = 175.4 ±51.5 (P value = < 0.001). Triglycerides 108.9 ±51.5 (P value = < 0.001). HDL = 48.0 ±9.1 (P value = < 0.02). LDL = 103.3 ±25.5 (P value = < 0.001)	Isotretinoin did not cause a worsening of hearing thresholds but significantly increased total blood cholesterol, triglycerides, and LDL levels and caused a decrease in HDL levels.
Low-dose isotretinoin therapy and blood lipid abnormality: A case series with sixty patients.	2018	Observational Study	60	Low-dose isotretinoin (20 mg orally)	• Hyperlipidemia • Hypertriglyceridemia • Elevation of VLDL • Elevation of LDL • Hypercholesterolemia	Males with hyperlipidemia-28.12% • Females with Hyperlipidemia- 21.43% (*P* = 0.6869) . Hypertriglyceridemia present in- 16.67% of patients, Elevation of VLDL present in—11.67%, LDL in 10% and Hypercholesterolemia present in 5%.	Low-dose Isotretinoin causes a variable rise in various lipid profile parameters. Frequent monitoring of serum lipid profile parameters is needed and should be used with caution in individualswith risk factors like metabolic syndrome.
Results of Laboratory Monitoring in Patients Taking Isotretinoin for Acne	2021	Retrospective study	371	Low-dose isotretinoin (median dose = 30 mg)	• Baseline Alanine Aminotransferase (ALT) • Aspartate Aminotransferase (AST) • Total Cholesterol • Triglycerides (Tgs)	First follow-up—high levels of AST in 7 (1.9%) patients. Second follow-up figure doubled (14 [3.8%] patients) (*P*>.05). ALT at the first and second follow-ups—(47/371 [12.7%], 49/371 [13.2%], and 37/371 [10.0%], respectively) (*P*>.05). Cholesterol levels at baseline and at both the first and second follow-ups (40/331 [12.1%], 72/331 [21.8%], and 62/331 [18.7%], (*P* = .001). TGs- 5/320 [1.6%], 12/320 [3.8%], and 14/320 [4.4%] at baseline and at the first and second readings, respectively.	The serum lipid profile was affected more than liver enzymes in patients who were treated with isotretinoin. But the changes in the lipid profile were non-progressive and non-severe; therefore, isotretinoin can be administered with minimal concern for changes in these parameters.

In another prospective cohort study conducted in India, 50 patients diagnosed with moderate to severe acne between the age group of 15-45 years attending the dermatology department were treated with 20 mg of isotretinoin daily for 4 months. Blood samples were collected on day 0, 2nd week, 1 month, 2 months, 3 months, and 4 months. Isotretinoin (20 mg daily for 4 months) showed a statistically significant increase in cholesterol, triglycerides, and LDL at all intervals compared with baseline and above the normal limit with a substantial decrease in HDL levels, but concluded that treatment did not need to be interrupted.

A retrospective chart review by Yu Jeong Park *et al* showed that AST, ALT, and TG levels significantly changed between the 5th and 6th months when the total prescription period (60-180 days) and dose variables (600–3600 mg) were considered together. HDL levels also significantly changed between the 5th and 6th months. However, LDL and total cholesterol levels significantly changed between 1st and 2nd months.

Another similar study was conducted at the Department of Dermatology, Qassim University Medical City, Saudi Arabia, which included all acne patients treated with isotretinoin for at least 4 months. AST and ALT levels increased in 5.4% and 12.7% of patients, respectively. Of the patients, 3.9% had elevated AST levels at their last visit and 9% had elevated ALT levels. Compared with 6.5% of patients at baseline, 12.7% had increased triglyceride levels during their most recent visit. Compared to 10.5% of patients at baseline, 9% had increased total cholesterol during their most recent visit.

To measure HDL at Baseline And After Treatment, HDL % Change, LDL % Change, Total Cholesterol % Change, AST At Baseline And After Treatment, and ALT At Baseline And Treatment after Isotretinoin treatment, a cross-sectional study of 200 patients showed a significant increase in the lipid profile (LDL, TC, and TG) and liver enzymes (AST and ALT) after the initiation of oral isotretinoin therapy compared to the baseline (*P* < 0.05). However, the HDL levels significantly decreased after therapy (*P* < 0.05). These changes were linked to the genotypes and polymorphisms of rs7799039.

Another systematic review and meta-analysis was conducted by Young H. Lee *et al* performed laboratory monitoring of acne during isotretinoin therapy. According to the analysis, isotretinoin treatment is associated with a significant increase in laboratory values, but this increase is insufficient to categorize it as a high risk. Therefore, this study does not support monthly laboratory testing of standard oral doses of isotretinoin for treating acne.

A retrospective review of laboratory data of patients undergoing isotretinoin treatment for acne was carried out by Timothy J. Hansen *et al*. It was concluded that lipid profiles and liver function tests in healthy patients should be performed after 2 months of oral isotretinoin therapy. If the findings were normal, further testing was not required.

Yaldiz *et al* conducted a prospective cohort study to assess the auditory function and lipid levels in patients undergoing oral isotretinoin therapy for acne vulgaris. This study showed that isotretinoin did not worsen hearing thresholds. However, it significantly increased the levels of total blood cholesterol, triglycerides, and LDL and caused a decrease in HDL levels.

Sarkar *et al* conducted a clinic-based observational study of patients with various skin disorders in eastern India. The study concluded that low-dose isotretinoin caused a variable increase in the lipid profile compared to baseline levels. Therefore, frequent monitoring of lipid profiles is required and should be performed for high-risk patients.

Al-Haddab *et al* conducted a retrospective cohort study of the effects of isotretinoin on various laboratory parameters. The study concluded that the serum lipid profile of these patients was affected more than the liver enzymes. However, the changes were non-progressive and non-severe; therefore, isotretinoin should be administered with minimal concern.

## Discussion

Isotretinoin (Iso) is the drug of choice for acne vulgaris. The objective of this meta-analysis was to analyze the effect of isotretinoin treatment on lipid profiles and related parameters based on the available literature. Given the growing concerns regarding the long-term effects of isotretinoin on lipid parameters, it is crucial to monitor lipid profiles throughout the therapy to ensure patient safety and achieve the best therapeutic results. Several studies were systematically analyzed to provide a comprehensive understanding of the objectives mentioned above.

Studies on isotretinoin have shown alterations in serum lipid levels, elevated liver enzymes, and notably, increased CIMT. While the clinical significance of the latter is still not well studied, it can be considered an indication for monitoring the cardiovascular health of patients on iso therapy.^[23]^.

In a further study of lipid profiles, there was an increase in serum TG, LDL, and Total Cholesterol levels in those who take intermittent Iso therapy^[24]^. These changes may be unfavorable for those already at risk of developing metabolic syndrome, dyslipidemia, or atherosclerosis. However, it is important to note that these changes were non-progressive and posed insignificant threats to an overall healthy individual^[25,26]^, and it is recommended to monitor lipid profiles, specifically during the first and second months of oral iso therapy.^[26]^ Elevated serum AST and ALT levels were also observed. Similar to lipid level changes, they were not associated with increased morbidity and did not meet any criteria to be considered high risk.^[27,28]^

We also explored the genetic basis for the alterations in lipid profile, and found that there is a modulatory role of polymorphisms of the leptin gene, rs7799039 on the studied changes^[29]^ This calls for further pharmacogenomics research to identify people at risk of developing side effects of iso treatment.

Overall, the changes may not be clinically significant and are simply suggestive of regular monitoring of the above parameters. A baseline assessment of serum lipid profile and liver function tests followed by another set of laboratory investigations after 2 months of treatment should be performed^[19]^. It is important to consider individual patient characteristics, conduct regular laboratory monitoring, and assess the overall risk-benefit profile when prescribing isotretinoin for acne management.

## Conclusion

We aimed to determine the effect of isotretinoin on lipid profiles in its therapeutic use for dermatological disorders, most commonly Acne. Most included studies consistently reported changes in the serum LDL/HDL ratio with prolonged isotretinoin use. Precautions are necessary when prescribing isotretinoin to patients with pre-existing dyslipidemia, hypercholesterolemia, metabolic syndrome, or other cardiovascular risk factors. The long-term use of isotretinoin leads to an increase in the carotid artery intima-media thickness and transient alterations in lipid profile, characterized by increases in total cholesterol, triglycerides, and LDL-C levels. Therefore, it is essential to assess lipid parameters, including baseline assessment of serum lipid profile and liver function tests, 2 months after starting treatment with isotretinoin, and AST, ALT, and TG levels should be checked biannually.

The observed changes may vary depending on the study population, dosage, and treatment duration. Although these changes are typically reversible and do not pose significant long-term cardiovascular risks, regular monitoring of lipid parameters during isotretinoin therapy is advised to improve the therapeutic outcomes.

Healthcare providers should consider individual patient characteristics and tailor management strategies to minimize the potential adverse effects on lipid metabolism.

## Limitations

The dosage and duration of isotretinoin therapy varied. Some studies have shown a significant effect of elevated lipid levels with a low dose of isotretinoin, whereas others revealed an elevation only with a high dose of isotretinoin. Some studies had a limited study duration. Moreover, gender differences in the response to isotretinoin are unclear. Compliance with ethical standards was not available. It is also possible to witness errors in levels based on preexisting liver disease and the use of hepatotoxic or lipid-altering drugs. This warrants a clinical judgment to determine the monitoring frequency of such patients.

## Data Availability

No data available.

## Research registration unique identifying number (UIN)

1. Registry used - PROSPERO

2. Unique Identifying number or registration ID - CRD42023428164

3. Hyperlink to your specific registration (must be publicly accessible and will be checked) -https://www.crd.york.ac.uk/prospero/display_record.php?ID=CRD42023428164
